# Dietary Approaches to Stop Hypertension (DASH) dietary pattern is not associated with blood pressure in a cross-sectional sample of Australian primary schoolchildren

**DOI:** 10.1007/s00394-025-03696-9

**Published:** 2025-05-13

**Authors:** Anne-Sophie Van Dijck, Ewa A. Szymlek-Gay, Carley A. Grimes

**Affiliations:** 1 Faculty of Economics and Business, Ghent University, Ghent, Flanders Belgium; 2 Institute for Physical Activity and Nutrition (IPAN), School of Exercise and Nutrition Sciences, Deakin University, Geelong, VIC Australia

**Keywords:** Dietary approaches to stop hypertension (DASH), Blood pressure, Children, Diet, Australia

## Abstract

**Purpose:**

The beneficial effects of the Dietary Approaches to Stop Hypertension (DASH) to reduce blood pressure among adults are well established. However, whether this dietary pattern is also relevant for the control of blood pressure in children remains unknown. Therefore, this study aimed to evaluate adherence to the DASH dietary pattern and examine its association with blood pressure among Australian primary schoolchildren.

**Method:**

Cross-sectional data from 658 Australian children aged 8–12 years participating in the Salt and Other Nutrients In Children (SONIC) study were analyzed. One 24-hour diet recall was used to assess dietary intake. Systolic and diastolic blood pressure were measured with a digital automatic blood pressure machine. To assess adherence to the DASH dietary pattern a total DASH score (0–90) was created based on nine nutrient targets (protein, saturated fat, total fat, cholesterol, total fiber, sodium, potassium, magnesium, calcium). Multiple linear regression models were used to examine the association between the DASH score and blood pressure.

**Results:**

The mean total DASH score was 53.1 (SD 10.4) and was significantly different between boys (52.3) and girls (54.0) (*p* = 0.013). After controlling for covariates, no association between total DASH score and systolic or diastolic blood pressure was found.

**Conclusion:**

Adherence to the DASH dietary pattern was moderate and there was no association between the DASH dietary pattern and blood pressure among Australian children aged 8–12 years.

**Supplementary Information:**

The online version contains supplementary material available at 10.1007/s00394-025-03696-9.

## Introduction

Hypertension is one of the major causes of premature death worldwide [1]. Between 1990 and 2019, the number of adults affected by hypertension worldwide has doubled from 650 million to 1.3 billion [2]. In children the prevalence of hypertension has also increased in the last two decades [3, 4]. The meta-analysis of Song et al. [4] estimated that the global prevalence of hypertension among children under 19 years was 4.0% in 2019, with overweight or obese children at greater risk. It is important to control high blood pressure in children as high blood pressure tracks over the life course [5, 6] and is associated with both sub-clinical markers of cardiovascular disease (CVD) (e.g. left ventricular hypertrophy, increased pulse wave velocity, high carotid intima-media thickness) [7] and CVD events, including mortality in adulthood [8].

Some evidence shows that single nutrients (e.g. sodium and fat) and particular foods (e.g. fruit and vegetables) affect blood pressure among children [9–11], but the impact of diet quality on blood pressure is unclear. The longitudinal study of Moore et al. [12] with 95 preschool-aged children 3–6 years at baseline, with an annual follow-up of 8 years, reported that a higher intake of a combination of food groups (dairy, fruits and vegetables) led to greater reductions in systolic blood pressure in early adolescence compared to a high intake of single food groups (e.g. dairy or fruit and vegetables alone). In addition, the review of Casas et al. [13] describes that a healthy dietary pattern has more beneficial effects on blood pressure than the potential effects of single nutrients. Additionally, they highlight the importance of promoting healthy dietary habits as early as possible in children. In line with these results, it may be of greater value to examine the relationship between dietary patterns and blood pressure rather than the impact of single nutrients or single food groups.

The Dietary Approaches to Stop Hypertension (DASH) is one of the most well-known and effective dietary recommendations to lower blood pressure and prevent hypertension among adults [14]. This diet emphasizes consumption of wholegrains; vegetables; fruits; low-fat dairy; lean meats, poultry and fish; and nuts, seeds and legumes; and limited consumption of sweets and added sugars [15]. The diet was designed to target nutrients hypothesized to alter blood pressure levels (e.g. decreased saturated fat, sodium, cholesterol and increased calcium, magnesium, potassium and dietary fibre) [15]. Overall evidence about the effect of the DASH diet on children’s blood pressure is limited. Findings from three randomized controlled trials that have tested the effect of a DASH diet on blood pressure in adolescents [16, 17] or with combined effects of physical activity in younger children aged 5–8 years [18] have produced mixed findings. Longitudinal studies completed in healthy samples of school-aged children from the Netherlands [19] and Iran [20] have shown that higher adherence to a DASH dietary pattern is protective against high blood pressure, either reducing the incidence of developing hypertension [20] or leading to reductions in systolic blood pressure [19]. Among cross-sectional studies, findings are varied [21–23]. For example, an inverse association between adherence to a DASH dietary pattern and systolic blood pressure was shown in representative samples of healthy children from Iran aged 6–12 years [22] and the USA, however in the latter this was only observed in older children aged 14–18 years and not those aged 8–13 years [21]. Conversely, in a sample of Spanish children aged 5–16 years, recruited from a hospital setting, no association between a modified DASH score (where sodium was omitted) and blood pressure was reported [23]. The American Heart Association acknowledges the importance of modifying dietary intake for primordial prevention of hypertension [24] and a DASH-type diet is recommended as a reasonable strategy for the prevention of hypertension during childhood [25]. Further investigation into the effects of a DASH diet on blood pressure levels in different pediatric population groups is required to strengthen the existing limited evidence base. Therefore, this study aimed to examine the association between adherence to the DASH dietary pattern and blood pressure among Australian primary schoolchildren. Given that certain dietary factors targeted in the DASH diet (e.g. sodium) may have a more pronounced effect on blood pressure among children who are overweight or obese [26], we also examined if the association between a DASH dietary pattern and blood pressure was modified by body weight.

## Methods

### Study design and participants

**Fig. 1:**
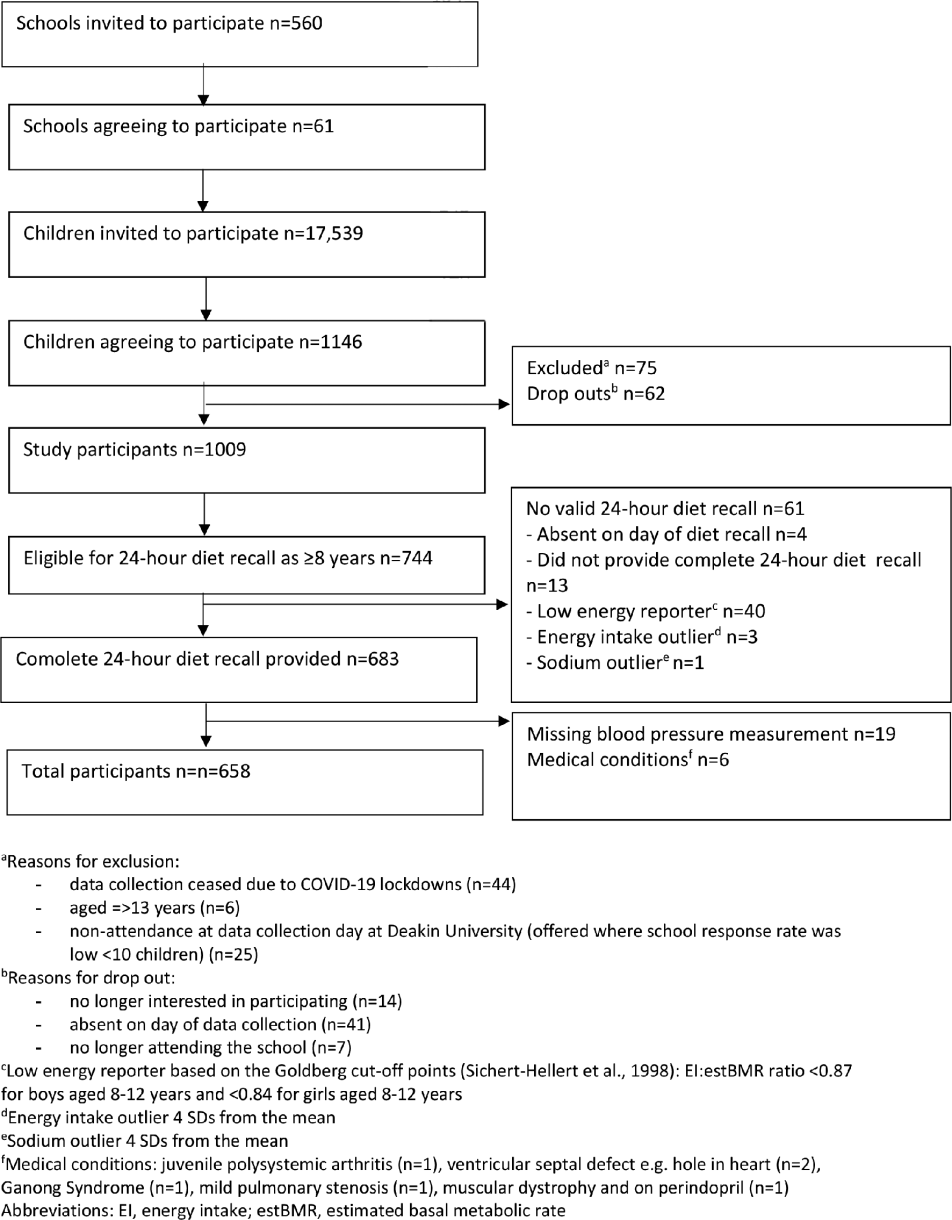
Flow chart of participants

This research utilized data from the previously described cross-sectional Salt and Other Nutrient Intakes in Children (SONIC) study of 4-12-year-old children from primary schools in Victoria, Australia, collected during two periods: 2010–2013 (timepoint 1) and 2018–2019 (timepoint 2) [27]. Briefly, a convenience sample of 61 schools participated, initially identified through an online school locator, with 509 schools invited at timepoint 1 and re-contacted at timepoint 2. An additional 51 schools were invited at timepoint 2 to maintain sample comparability. Invitation letters and study information were sent to parents via children (*n* = 17,539), resulting in 1146 participants. Of these, 744 children met the age requirement for the current study (≥ 8 years). Exclusions were made for children absent on the day of the recall (*n* = 4), those with invalid dietary recalls (*n* = 57), missing blood pressure data (*n* = 19), or existing health conditions affecting blood pressure (*n* = 6). This yielded a final sample of 658 children for the current analysis (Fig. 1).

The protocol of the SONIC study was approved by Deakin University Ethics Committee (ID numbers: EC62-2009 and HEAG-H 01_2018). The Victorian Department of Education and Training gave permission to conduct this research in government schools (ID numbers: 2011_001151 and 2018_003666). In non-government schools’ permission was granted by the school board or the principal. School principals and parents granted informed written consent to participate, written assent was obtained from the children.

### Sample characteristics

Information on demographic characteristics for children and parents was collected through a questionnaire completed by the parents. Further information on the demographic information can be found in earlier published research of the SONIC study [28]. The weight and height of the child were measured following standard protocols [27] with a calibrated portable electronic scale (FS-127-BRW, Bradman, MA, USA) and portable stadiometer (SECA mod 220, Hamburg, Germany), respectively. BMI was calculated as body weight (kg) divided by the square of body height (m^2^). BMI values were converted to age- and sex-adjusted BMI z-score using the 2000 US Centers for Disease Control and Prevention growth charts [29] and classified as underweight, healthy weight, overweight or obese [30, 31]. The Index of Relative Socio-economic Disadvantage was used to describe socio-economic status for each child based on the postcode of where the child attended school [32].

### Blood pressure

Blood pressure was measured using a digital automatic blood pressure machine (OMRON HEM-907) after 10 min of rest. Three measurements were taken on the right arm with a 1-minute interval between each measurement. The mean of the second and third measures was used in the final analysis. Systolic (SBP) and diastolic (DBP) blood pressure percentiles adjusted for sex, age and height were calculated using the 2017 American Academy of Pediatrics (AAP) Guidelines [25]. Blood pressure z-scores (SBP and DBP) were obtained from the blood pressure percentiles using the inverse normal distribution. Normal blood pressure was defined as SBP and DBP < 90th percentile, and elevated blood pressure as SBP or DBP ≥ 90th percentile [25].

### Diet intake data

Dietary intake was assessed using one 24-hour dietary recall, where the child recalled all food and beverage consumption over the previous 24-hour period to a trained research assistant. At timepoint 1, the dietary recall information was entered into a nutrient analysis program FoodWorks (Xyris, Brisbane) to obtain nutrient intakes. At timepoint 2, the ASA-24-Australia-2016, a newly developed web-based 24-hour dietary assessment tool [33], was used to directly input food recall information to obtain nutrient intakes. At both timepoints, the 24-hour food and beverages intakes were converted into nutrient intakes using the Australian food composition database AUSNUT 2011–2013 [34]. Diet recalls were excluded if the participant did not complete a 24-hour diet recall (*n* = 57), their energy intake or sodium intake was considered as an outlier (4SD from the mean), or they were deemed as a low-energy intake reporter (Fig. 1). A low energy reporter was based on the Goldberg cut-off values whereby each child’s ratio of reported energy intake (EI) to estimated basal metabolic rate (BMR) was compared to the pediatric adjusted Goldberg cut-off value (the EI: estBMR ratio cut off value was < 0.87 for boys and < 0.84 for girls) [35].

### DASH score

To assess adherence to the DASH dietary pattern, an age-appropriate nutrient-based DASH score, which was based on the method of Cohen et al. [21], with some modifications, was calculated. The nutrient-based DASH score included targets for nine nutrients: total fat, saturated fat, protein, fibre, cholesterol, sodium, potassium, magnesium and calcium. For each nutrient a target was set based on the DASH dietary pattern for adults [36] and adapted to children. Specifically, the targets for protein, total fat, and saturated fat were expressed as a proportion of total daily energy consumed from these nutrients, which directly aligned with the original targets specified for each of these nutrients in the DASH adult diet (Supplementary Table 1). For cholesterol, we used a target of 200 mg/day, which aligned with the target recommended by the AAP for lowering serum lipids in children with a high risk of cardiovascular disease [37]. For the other remaining five nutrients, the original DASH dietary pattern targets for adults, which aligned closely with the US adult nutrient reference values, were replaced with child-specific Australian Nutrient Reference Values: for fibre and potassium the targets were based on the Adequate Intake, for calcium and magnesium the targets were based on the Recommended Dietary Intake, for sodium the target was based on the Upper Level (Supplementary Table 1) [38].

For protein, total fibre, potassium, magnesium and calcium, a score of 0–10 points was applied. Participants achieved ten points when they met the target for the nutrient. Intakes below the target were scored in a proportionate way as follows: the ratio of the participant’s daily nutrient intake to the target of the nutrient was multiplied by ten, (e.g. if a 9-year-old boy consumed 200 mg/day of magnesium, this intake was divided by the magnesium target of 240 mg/day and multiplied by ten: 200 mg/day/240 mg/day x10 = 8.33 points). For sodium, total fat, saturated fat and cholesterol, the ratio method was not used because it was deemed inappropriate to assign any points to nutrients known to be detrimental to health when consumed in excess. As such, for these targets, participants received zero points if they were above the target or ten points if their intake was under the target. To create the total DASH score, the score of the nine nutrient targets was summed for a possible range of 0–90, a score of 90 represented a dietary pattern perfectly aligned with the DASH dietary pattern recommendations.

A sensitivity analysis was conducted, in which an alternative DASH diet score was calculated using Cohen et al.’s [21] method whereby, one point was allocated for meeting a DASH target for a nutrient or zero points were allocated for not meeting the target. The final score had a possible range of 0–9 points, with a score of nine indicating a dietary pattern perfectly aligned with the DASH dietary pattern.

### Statistical analysis

Data were analyzed using STATA SE (version 17, StataCorp LP, College Station, TX). The datasets of the two timepoints were combined, because there was no significant difference in mean total DASH score between each timepoint (adjusted for age, sex, socio-economic status, diet recall day and BMI z-score, p-value = 0.10). Descriptive statistics were used to describe sample characteristics, nutrient intakes, and total DASH scores. Data are presented overall and stratified by sex. Independent sample t-test was used to examine if the total DASH score differed between boys and girls. To examine the association between the total DASH score (independent variable) and SBP and DBP (dependent variables), multiple linear regression models were used. Models were completed using raw blood pressure values (mm Hg) as well as calculated blood pressure z-scores. Both unadjusted and adjusted models are presented. The final model was adjusted for age, sex, BMI z-score, diet recall day (i.e. school day vs. non-school day) and socio-economic status. In all models the clustering of participants within schools was accounted for. A p-value < 0.05 was considered statistically significant. Interaction terms of total DASH score with BMI weight category (grouped as underweight/healthy weight vs. overweight/obese) were added within each of the regression models to determine if the association between total DASH score and blood pressure was modified by weight. The Wald test was used to check overall interaction effects within the models. Findings stratified by weight category are presented.

## Results

### Participant characteristics

A total of 658 children were included in the current study, of which just over half were boys (Table 1). The mean age of the participants was 10.2 (SD 1.2) years. Most children (73%) were categorized in the healthy weight category and 80% of the children had normal blood pressure (Table 1).

**Table 1:** Socio-demographic characteristics of participants overall and by sex

	Overall (*n* = 658)			Girls (*n* = 306, 46%)			Boys (*n* = 352, 54%)		
	*n*	Mean or %	SD	*n*	Mean or %	SD	*n*	Mean or %	SD
Age (y)		10.2	1.2		10.1	1.2		10.3	1.2
Weight (kg)		37.1	9.3		37.2	9.3		37.1	9.3
Height (cm)		142.6	9.5		143.4	9.3		142.8	9.6
BMI z-score		0.22	0.98		0.22	0.99		0.22	0.97
BMI categories^a^									
Underweight Healthy weight Overweight Obese	45 483 107 23	7% 73% 16% 4%		24 216 56 10	8% 71% 18% 3%		21 267 51 13	6% 76% 14% 4%	
Diet recall day									
School day Non-school day	168 490	25% 75%		80 226	26% 74%		88 264	25% 75%	
Parental educational attainment^b^									
								21% 17% 62%	
Low Medium High Missing	118 83 344 113	22% 15% 63%		55 33 158 60	22% 14% 64%		63 50 186 53		
Level of socio-economic disadvantage^c^									
								20% 20% 60%	
Most disadvantaged Medium level of disadvantage Least disadvantaged	146 154 358	22% 24% 54%		75 82 149	24% 27% 49%		71 72 209		
Blood pressure categories ^d^									
								79% 21%	
Normal (SBP and DBP < 90th percentile) Elevated (SBP or DBP ≥ 90th percentile)	527 131	80% 20%		248 58	81% 19%		279 73		
SBP (mm Hg)		104.1	10.2		103.5	10.4		104.7	10.0
SBP percentile		58.4	27.9		56.9	28.8		59.8	27.0
SBP z-score		0.28	1.0		0.24	1.0		0.32	0.95
DBP (mm Hg)		61.3	8.8		61.6	8.5		61.1	9.1
DBP percentile		50.1	25.6		52.0	25.7		48.4	25.4
DBP z-score		0.03	0.8		0.09	0.8		-0.02	0.8

Abbreviations: BMI, body mass index; DBP, diastolic blood pressure; SBP, systolic blood pressure,

^a^ Based on the international Obesity Task Force BMI reference cut-offs [30, 31]

^b^ low: some or no high school education; medium: technical/trade certificate; high: university/tertiary qualification

^c^ Tertiles of the Index of Relative Socio-economic Disadvantage based on children’s school postcodes [32]

^d^ Based on the 2017 American Academy of Pediatric Guidelines [25]

### Dietary intake

**Fig. 2:**
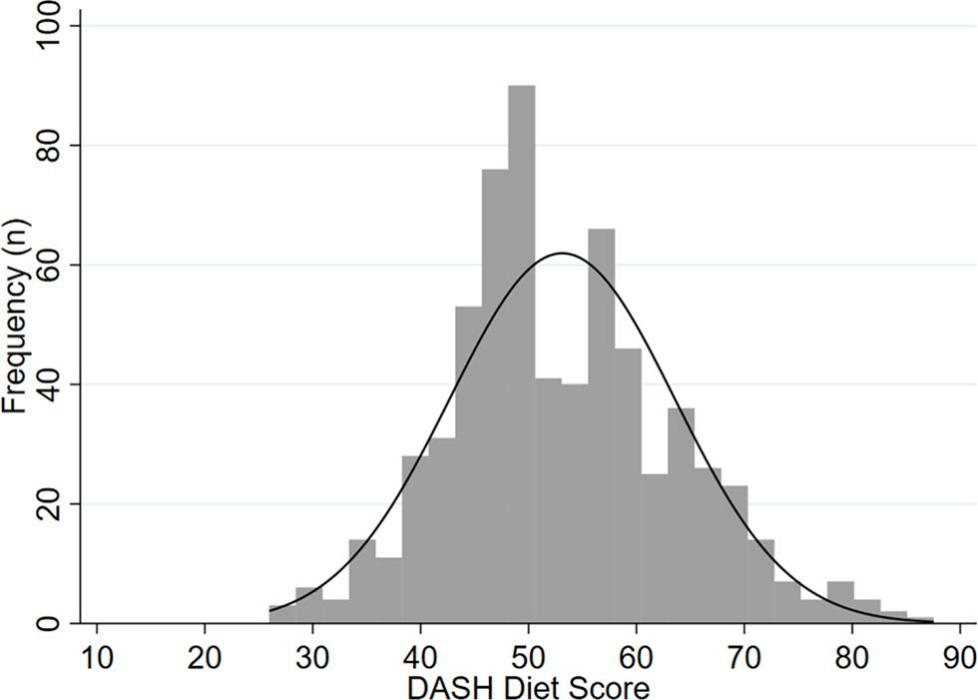
Distribution of the total DASH scores among children aged 8–12 years (*n* = 658)^a^. ^a^ Curved line represents normal distribution. Abbreviation: DASH Dietary Approaches to Stop Hypertension

The mean energy intake among the children was 8430 (SD 2574) kJ/d (Table 2). About half (49.6 (SD 8.1) % kJ/day) of the daily energy intake consisted of carbohydrate. The mean total DASH score among the participants was 53.1 out of 90 (SD 10.4, min 26.0, max 87.5), which was indicative of moderate adherence to the DASH dietary pattern (Fig. 2). There was a significant difference in total DASH score between boys (means score 52.3; SD 10.3) and girls (mean score 54.0; SD 10.5) (mean difference − 1.7; p-value = 0.013). Total DASH score was also significantly higher on a school day (mean score 54.1; SD 10.1) vs. a non-school day (mean score 50.1; SD 10.9) (mean difference 4.0; p-value < 0.001). The targets that were achieved by at least half of children were magnesium and cholesterol, with 63% and 51% of the children meeting the target, respectively (Table 3). Only 3% of the participants had a saturated fat intake lower than the target of 6% kJ/d (Table 3).

**Table 2:** Dietary intake of children aged 8–12 years, overall and by sex

	Total (*n* = 658)		Girls (*n* = 306)		Boys (*n* = 352)	
	Mean	SD	Mean	SD	Mean	SD
Energy (kJ/d)	8430	2574	8015	2357	8789	2700
Macronutrients (g/d)						
Carbohydrates	250.1	81.3	238.1	76.5	260.4	84.0
Protein	78.5	29.6	74.3	27.6	82.1	30.9
Total fat	73.4	32.3	69.7	28.0	76.7	35.3
Saturated fat	30.4	14.2	29.4	14.2	31.3	14.3
Macronutrients (% kJ/d)						
Carbohydrates	49.6	8.1	49.8	7.8	49.9	8.5
Protein	15.7	4.1	15.6	3.9	15.7	4.3
Total fat	32.3	7.6	32.4	7.1	32.2	7.9
Saturated fat	13.4	4.1	13.5	4.2	13.3	4.0
Cholesterol (mg/d)	240.7	170	224.0	146.6	255.3	187.0
Dietary fibre (g/d)	22.1	9.8	21.4	8.9	22.8	10.4
Potassium (mg/d)	2489.6	894.6	2406.5	886.8	2561.8	896.3
Calcium (mg/d)	809.9	428.4	783.5	393.9	832.9	455.6
Magnesium (mg/d)	268.7	99.9	255.1	83.7	280.5	111.0
Sodium (mg/d)	2410.9	1067.2	2310.9	1007.3	2497.9	1110.8

**Table 3:** Number of children aged 8–12 years meeting DASH nutrient targets and the mean DASH score for each nutrient target, overall and by sex

DASH components	Total (*n* = 658)		Girls (*n* = 306)		Boys (*n* = 352)	
	Meeting DASH target, *n* (%)	Mean DASH score out of 10 (SD)	Meeting DASH target, *n* (%)	Mean DASH score out of 10 (SD)	Meeting DASH target, *n* (%)	Mean DASH score out of 10 (SD)
Total fat	169 (26%)	2.6 (4.4)	71 (23%)	2.3 (4.2)	98 (28%)	2.8 (4.5)
Saturated fat	22 (3%)	0.3 (1.8)	11 (4%)	0.4 (1.9)	11 (3%)	0.3 (1.7)
Protein	170 (26%)	8.2 (1.5)	78 (25%)	8.3 (4.1)	92 (26%)	8.3 (4.1)
Fibre	300 (46%)	8.5 (1.8)	152 (50%)	8.7 (1.7)	148 (42%)	8.4 (1.9)
Cholesterol	335 (51%)	5.1 (5.0)	166 (53%)	5.4 (5.0)	169 (48%)	4.8 (5.0)
Sodium	228 (35%)	3.5 (4.8)	122 (40%)	4.0 (4.9)	106 (30%)	3.0 (4.6)
Potassium	259 (39%)	8.3 (1.9)	141 (46%)	8.5 (1.9)	118 (34%)	8.2 (2.0)
Magnesium	416 (63%)	9.3 (1.2)	180 (59%)	9.2 (1.3)	236 (67%)	9.4 (3.5)
Calcium	218 (33%)	7.3 (2.6)	106 (35%)	7.3 (2.7)	112 (32%)	7.3 (2.6)

Abbreviations: DASH, Dietary Approaches to Stop Hypertension

### Associations between total DASH score and blood pressure

No associations were found between the DASH score and any of the measures of blood pressure (raw SBP (mm Hg), raw DBP (mm Hg), SBP z-score, or DBP z-score) in the overall sample, or when stratified by sex (Table 4). An overall significant interaction effect was found between total DASH score and weight category (underweight/healthy weight vs. overweight/obese) with SBP (mm Hg) (Wald test p-value = 0.03); DBP (mm Hg) (Wald test p-value = 0.02) and DBP z-score (Wald test p-value = 0.02). The p-value for the Wald test interaction for SBP z-score was 0.057. However, findings stratified by weight category showed there was no association between total DASH score and any of the blood pressure outcomes in children who were either underweight/healthy weight or overweight/obese (Table 5).

**Table 4:** Associations of total DASH score and blood pressure among children aged 8–12 years overall and by sex (*n* = 658)

Outcome variable	Overall (*n* = 658)		Girls (*n* = 306)		Boys (*n* = 352)	
	b-coefficient (95% CI); *p*-value	*R*^2^; *p*-value	b-coefficient (95% CI); *p*-value	*R*^2^; *p*-value	b-coefficient (95% CI); *p*-value	*R*^2^; *p*-value
**SBP (mm Hg)**						
Unadjusted	-0.004 (-0.08, 0.07); 0.92	0.000; 0.92	-0.01 (-0.11, 0.10); 0.87	0.0001; 0.87	0.01 (-0.10, 0.12); 0.85	0.0001; 0.85
Adjusted^a^	0.03 (-0.04, 0.10); 0.35	0.09; <0.001	-0.00007 (-0.09, 0.08); 0.99	0.08; <0.001	0.06 (-0.05, 0.20); 0.28	0.11, < 0.001
**DBP (mm Hg)**						
Unadjusted	-0.01 (-0.08, 0.06); 0.76	0.0002; 0.76	0.004 (-0.09, 0.10); 0.94	0.000; 0.93	-0.03 (-0.13, 0.08); 0.60	0.000; 0.60
Adjusted^a^	0.02 (-0.05, 0.08); 0.62	0.06; <0.001	0.02 (-0.08, 0.12); 0.68	0.06; <0.001	0.01 (-0.08, 0.10); 0.80	0.08; <0.001
**SBP z-score**						
Unadjusted	0.001 (-0.01, 0.01); 0.74	0.0001; 0.74	0.001 (-0.01, 0.01); 0.84	0.0001; 0.84	0.002 (-0.01, 0.01); 0.65	0.005; 0.65
Adjusted^a^	0.002 (-0.05, 0.01); 0.62	0.01; 0.55	-0.002 (-0.01, 0.01); 0.96	0.01; 0.65	0.003 (-0.01, 0.01); 0.48	0.01; 0.43
**DBP z-score**						
Unadjusted	0.0002 (-0.01, 0.01); 0.95	0.000; 0.95	0.001 (-0.01, 0.01); 0.82	0.0002; 0.83	-0.001 (-0.01, 0.08); 0.74	0.0003; 0.74
Adjusted^a^	0.001 (-0.004, 0.007); 0.67	0.000; 0.03	0.002 (-0.01, 0.01); 0.73	0.04; 0.0001	0.001 (-0.01, 0.01); 0.86	0.04; 0.0001

Abbreviations: CI, confidence interval; DBP, diastolic blood pressure; SBP, systolic blood pressure

^a^Adjusted for age, sex, level of socio-economic disadvantage, diet recall day, BMI z-score; sex was omitted from sex stratified models

**Table 5:** Associations of total DASH score and blood pressure among children aged 8–12 years stratified by weight category^a^

Outcome variable	Underweight & healthy weight (*n* = 528)		Overweight & obese (*n* = 130)	
	b-coefficient (95% CI); *p*-value	*R*^2^; *p*-value	b-coefficient (95% CI); *p*-value	*R*^2^; *p*-value
SBP (mm Hg)	0.06 (-0.02 to 0.14); 0.16	0.08; <0.001	-0.13 (-0.32 to 0.07); 0.20	0.10; 0.001
DBP (mm Hg)	0.04 (-0.03 to 0.11); 0.25	0.04; <0.001	-0.14 (-0.28 to 0.004); 0.06	0.09; <0.001
SBP z-score	0.004 (-0.003 to 0.01); 0.28	0.006; 0.82	-0.01 (-0.03 to 0.009); 0.26	0.05; 0.04
DBP z-score	0.003 (-0.003 to 0.009); 0.25	0.02; <0.001	-0.01 (-0.03 to 0.002); 0.08	0.07; <0.001

Abbreviations: CI, confidence interval; DBP, diastolic blood pressure; SBP, systolic blood pressure

^a^Models are adjusted for age, sex, level of socio-economic disadvantage and diet recall day

Sensitivity analysis results appeared similar. The adherence to the DASH dietary pattern based on the alternative DASH score was low with the mean score of 3.0 out of 9 (SD 1.5; min 0.0, max 7.0) (Supplementary Fig. 1). For boys, the mean alternative DASH score was 2.9 (SD 1.6) and for girls it was 3.2 (SD 1.5) (p-value = 0.03 for the difference between boys and girls). Supplementary Table 2 presents the results of the regression models examining the association between the alternative DASH score and blood pressure, which were consistent with the main analysis.

## Discussion

This was the first study to evaluate the adherence to the DASH dietary pattern and examine the association between the DASH dietary pattern and blood pressure among Australian children aged 8–12 years. The adherence to the DASH dietary pattern was moderate and no association was found between the DASH dietary pattern and blood pressure. Furthermore, the association between the DASH dietary pattern and blood pressure was not modified by body weight.

The mean adherence to the DASH dietary pattern was moderate with none of the children achieving the highest possible score of 90. Overall, the dietary intake of the children in this study mirrored that reported in the 2011–2012 Australian Health Survey [39]. Our study showed a higher adherence to the DASH dietary pattern than what has been observed among children from the USA [21, 40]. For example, Cohen et al. reported a mean dash score that ranged from 1.48 to 2.14 among children aged 8–18 years who participated in the National Health and Nutrition Examination Survey. This is lower than what we reported in the current study, when using the same scoring system as Cohen et al. e.g. 3.4 out of 9. Comparing our findings with other studies poses challenges due to variations in the assessment of dietary intake e.g. 24-hour diet recall vs. food frequency questionnaire (FFQ) and scoring methods used to determine adherence to the DASH dietary pattern. For example, Krijger et al. [19] and Günther et al. [40] used food groups to determine adherence in Dutch and US children, whereas our study used nutrient targets.

The DASH dietary pattern was not associated with blood pressure in the current study. This aligns with findings from Pérez-Gimeno et al. [23], who conducted a cross-sectional study involving 687 Spanish children and adolescents aged 5–16 years. Of note, Pérez-Gimeno et al.’s method for assessing adherence to the DASH dietary pattern differed from ours, as they based the total DASH score on food groups. Similar to our study, the study by Cohen et al. [21] involving a large sample of 8-18-year-old US children (*n* = 9,793) reported no association between the DASH score and SBP and DBP based on a single 24-hour diet recall. However, in analyses stratified by age group the authors did observe a small inverse association between the DASH score and SBP among adolescents aged 14–18 years. In sensitivity analyses, the authors also reported a significant inverse association between DASH score and SBP among children aged 11–13 years when data from 2 days of 24-hour dietary recall was considered [21]. No association among younger children aged 8–10 years was found.

Other cross-sectional [22] and longitudinal studies [20, 22] have reported that a DASH diet can be beneficial for maintaining healthy blood pressure during childhood. These studies have utilized FFQs to collect dietary information and food groups to assess adherence to the DASH dietary pattern. In a cross-sectional study of Iranian primary school children aged 6–12 years, Najafi et al. [22] found that a greater adherence to the DASH score was associated with significantly lower blood pressure, whereby the difference in SBP was − 6 mm Hg, *p* = 0.01 between participants within the highest vs. the lowest tertile of DASH score, however no association was found for DBP. In a longitudinal study, Krijger et al. [19] found a significant association between the DASH dietary pattern and blood pressure in 869 Dutch primary school children. Specifically, higher DASH scores at age 5–6 years were associated with lower SBP (-2.4 mm Hg, *p* = 0.046) and DBP at age 11–12 years (-2.4 mm Hg, *p* < 0.001). Finally, in a longitudinal study of Iranian children aged 6–18 years (mean age 13.6 years), the incidence of developing hypertension after 3.6 years of follow-up was significantly reduced among those in the highest quartile of DASH score vs. those in the lowest quartile [20]. It is unclear if the age of children or methods used to assess dietary intake or adherence to the DASH dietary pattern may account for inconsistencies in results observed across studies and additional investigation is warranted.

In our study the association between the DASH dietary pattern and blood pressure was not modified by body weight. To our knowledge, no previous studies in children have examined the impact of weight on the association between the DASH dietary pattern and blood pressure. Further research is needed to investigate how DASH dietary factors or patterns affect blood pressure and how this impact varies among children living with overweight or obesity.

The DASH dietary pattern is comprised of nine nutrients. These nutrients have an impact on the blood pressure via various mechanisms. Sodium exerts its influence on blood pressure via multifaceted mechanisms encompassing fluid balance, renal function, hormonal regulation, inflammatory responses, and alterations to the gut microbiome [41]. The DASH dietary pattern is characterized by the consumption of foods such as fruits, vegetables, whole grains and dairy products, which are abundant in calcium, magnesium, potassium, and antioxidants. These nutrients have been demonstrated to reduce blood pressure by promoting vasodilation and supporting enhanced regulation of the cardiovascular system [42, 43]. Additionally, these foods are high in dietary fibre. Dietary fibre, in particular, has been shown to reduce blood pressure and decrease cardiovascular risk, likely through the influence of short-chain fatty acids derived from gut microbiota. These short-chain fatty acids act via multiple biological pathways to affect blood pressure [44].

The present study has several strengths. A 24-hour diet recall was used to assess dietary intake, which allowed for collecting detailed information about the dietary intake of the children. The use of 24-hour diet recall allows for detailed information on all food and drinks consumed. In addition, we also had a large sample size. Standard protocols were used for measuring blood pressure, weight, and height. Limitations of the study should be noted. Recall bias may have impacted the collection of dietary intake data. The reliance on a single day of dietary recall precluded the estimation of the children’s usual intake, limiting the ability to observe associations between dietary patterns and blood pressure [45]. To assess usual intakes, two 24-hour diet recall are needed for at least a subset of participants [46]. The software used to code dietary intake differed between timepoint 1 and timepoint 2. However, both software programs were underpinned by the same food composition database, and no significant differences in DASH scores were found between the two timepoints. The developed DASH adherence score was based on nutrient targets that aligned with the original DASH diet, rather than the corresponding DASH diet food groups [15]. This may overlook the holistic effects of dietary patterns on blood pressure. Findings may not be generalizable to populations outside of Australia, where dietary habits and blood pressure determinants vary. The data collected were cross-sectional, which cannot give information about the causal effect of the DASH dietary pattern on blood pressure. Lastly, no data were collected about relevant covariates such as physical activity or sedentary behavior, which may confound the relationship between dietary patterns and blood pressure.

In conclusion, we found that Australian children aged 8–12 years had moderate adherence to the DASH dietary pattern. No significant association was observed between the DASH dietary pattern and blood pressure and this association was not modified by body weight. Further research is needed to understand the association between the DASH dietary pattern and blood pressure among primary school children. Specifically, future research should explore this association across different age and weight groups and over the long term. Further longitudinal data are necessary to establish the causal effect of the DASH dietary pattern on blood pressure among children.

## Electronic supplementary material

Below is the link to the electronic supplementary material.

Supplementary Material 1

## Data Availability

The data used in this study cannot be made publicly available as participants did not provide consent for their data to be used for purposes other than described in the original study aims.
