# Supplementary material for: Dietary Approaches to Stop Hypertension (DASH) dietary pattern is not associated with blood pressure in a cross-sectional sample of Australian primary schoolchildren
Source: Eur J Nutr. 2025 May 13;64(4):178. doi: 10.1007/s00394-025-03696-9 (PMC12075353; doi:10.1007/s00394-025-03696-9)
Supplement: Supplementary file 1 — Supplementary Material 1 [file 394_2025_3696_MOESM1_ESM.docx]

**Supplementary Table 1** Scoring system used to calculate DASH score

| **Nutrient** | **DASH original target for adults [1]** | **DASH target as per Cohen et al.[2]** | | | **DASH target for Australian children** | | **Rationale** |
| --- | --- | --- | --- | --- | --- | --- | --- |
|  |  | **8-10 years** | | **11-13 years** | **8-years** | **9-12 years** |  |
| Total fat | 27% of energy | ≤27% of energy | | | ≤27% of energy | | Consistent with the energy targets in the DASH adult diet [1]. |
| Saturated fat | 6% of energy | ≤6% of energy | | | ≤6% of energy | | Consistent with the energy targets in the DASH adult diet [1]. |
| Protein | 18% of energy | ≥18% of energy | | | ≥18% of energy | | Consistent with the energy targets in the DASH adult diet [1]. |
| Cholesterol | 150 mg/d | ≤150 mg/d | | | ≤200 mg/d | | Scoring based on 200 mg/day, an individual approach to lowering serum lipids for children with high risk for cardiovascular disease[3]. |
| Magnesium | 500 mg/d | ≥240 mg/ | | | ≥130 mg/d | ≥240 mg/d | Similar to Cohen et al. [2] target based on the age-specific Australian RDI for children[4]. |
| Sodium | 2300 mg/d | ≤2300 mg/d | | | ≤1400 mg/d | ≤2000 mg/d | Similar to Cohen et al. [2] based on age-specific Australian UL for children [4]. |
| Fibre | 30 g/d | ≥25 g/d | | ≥31 g/d (M)  ≥26 g/d (F) | ≥18 g/d | ≥24 g/d (M)  ≥20 g/d (F) | Similar to Cohen et al. [2] target based on age and sex-specific Australian AI for children [4]. |
| Calcium | 1250 mg/d | ≥1000 mg/d | | ≥1300 mg/d | ≥700 mg/d | 9-11 y: ≥1000 mg/d  12 y: ≥1300 mg/d | Similar to Cohen et al. [2] target based on the age and sex-specific Australian RDI for children [4]. |
| Potassium | 4700 mg/d | ≥3800 mg/d | ≥4500 mg/d | | ≥2300 mg/d | ≥3000 mg/d (M)  ≥2500 mg/d (F) | Similar to Cohen et al. [2] target based on Australian age and sex-specific AI for children [4]. |

Abbreviations: AI, Adequate Intake; DASH, Dietary Approaches to Stop Hypertension; F, female; M, male; NHLBI, National Hart, Lung and Blood Institute; NHMRC, National Health and Medical Research Council; NRV, Nutrient Reference Values; RDI, Recommended Daily Intake; UL, Upper Level

**Supplementary Figure 1** Distribution of alternative DASH scores among children aged 8-12 years (n=658)^1^

*
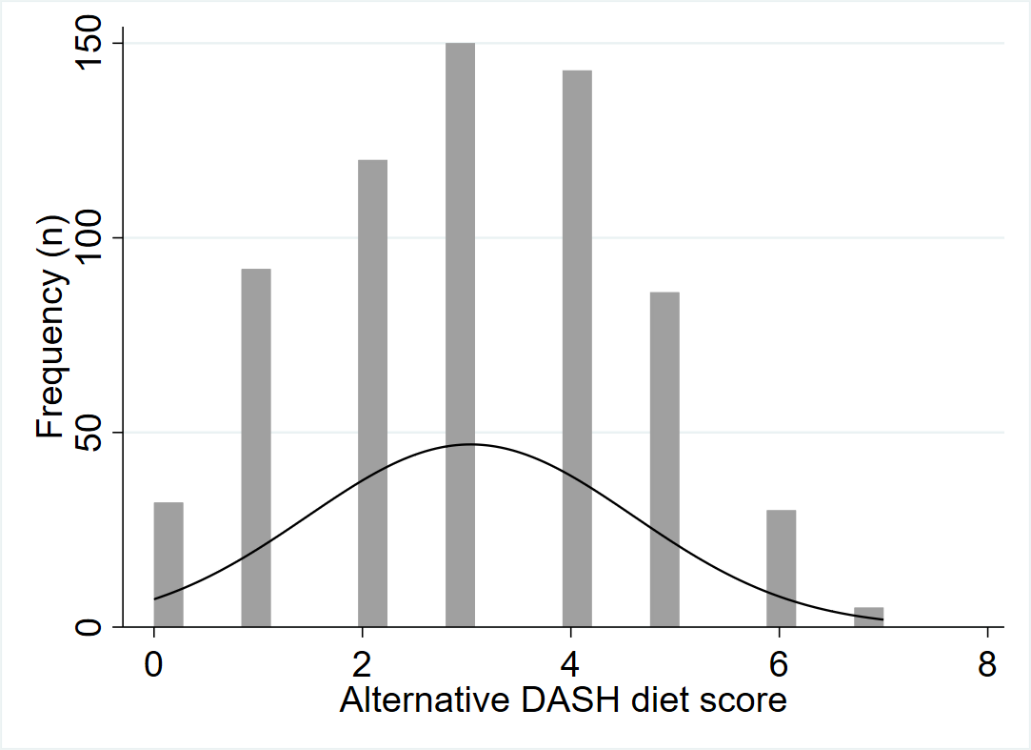
*

^1^ Curved line represents normal distribution

Abbreviation: DASH Dietary Approaches to Stop Hypertension

**Supplementary Table 2** Associations between alternative DASH score and blood pressure among children aged 8-12 years overall and by sex

| **Outcome variable** | **Overall (n=658)** | | **Girls (n=306)** | | **Boys (n=352)** | |
| --- | --- | --- | --- | --- | --- | --- |
|  | **b-coefficient (95% CI); p-value** | **R^2^; p-value** | **b-coefficient (95% CI); p-value** | **R^2^, p-value** | **b-coefficient (95% CI); p-value** | **R^2^, p-value** |
| **SBP (mm Hg)** |  |  |  |  |  |  |
| Unadjusted | -0.18 (-0.80, 0.39); 0.52 | 0.001; 0.52 | 0.04 (-0.63, 0.71); 0.90 | 0.000; 0.90 | -0.32 (-1.11, 0.47); 0.41 | 0.03; 0.41 |
| Adjusted^a^ | 0.17 (-0.4, 0.7); 0.53 | 0.09; <0.001 | 0.24 (-0.38, 0.86); 0.44 | 0.08; <0.001 | 0.11 (-0.69, 0.92); 0.78 | 0.11; <0.001 |
| **DBP (mm Hg)** |  |  |  |  |  |  |
| Unadjusted | -0.32 (-0.76, 0.11); 0.14 | 0.003; 0.14 | -0.21 (-0.77, 0.34); 0.45 | 0.002; 0.45 | -0.44 (-1.07, 0.19); 0.17 | 0.006; 0.17 |
| Adjusted^a^ | -0.07 (-0.49, 0.35); 0.73 | 0.06; <0.001 | -0.02 (-0.61, 0.57); 0.94 | 0.06; 0.0001 | -0.11 (-0.68, 0.46); 0.69 | 0.08; <0.001 |
| **SBP z-score** |  |  |  |  |  |  |
| Unadjusted | -0.003 (-0.06, 0.05); 0.90 | 0.000, 0.90 | 0.01 (-0.05, 0.08); 0.64 | 0.001; 0.64 | -0.02 (-0.09, 0.06); 0.68 | 0.001; 0.68 |
| Adjusted^a^ | 0.003 (-0.05, 0.06); 0.91 | 0.01; 0.48 | 0.01 (-0.05, 0.07); 0.65 | 0.01; 0.66 | -0.05 (-0.08, 0.07); 0.90 | 0.01; 0.46 |
| **DBP z-score** |  |  |  |  |  |  |
| Unadjusted | -0.02 (-0.06, 0.02); 0.40 | 0.001; 0.39 | -0.02 (-0.07, 0.04); 0.55 | 0.0009; 0.55 | -0.02 (-0.07, 0.03); 0.41 | 0.002; 0.41 |
| Adjusted^a^ | -0.01 (-0.05, 0.03); 0.73 | 0.03; <0.001 | -0.007 (-0.06, 0.05); 0.81 | 0.04; <0.001 | -0.007 (-0.06, 0.04); 0.79 | 0.04; <0.001 |

*Abbreviations: CI, confidence interval; DBP, diastolic blood pressure; SBP, systolic blood pressure*

^a^Models are adjusted for age, sex, level of socio-economic disadvantage and diet recall day; sex omitted from sex stratified models

**References**

1. NHBLI. DASH eating plan <https://www.nhlbi.nih.gov/files/docs/public/heart/new_dash.pdf>. Accessed 20 June 2024.

2. Cohen JFW, Lehnerd ME, Houser RF, Rimm EB. Dietary Approaches to Stop Hypertension Diet, Weight Status, and Blood Pressure among Children and Adolescents: National Health and Nutrition Examination Surveys 2003-2012. *J Acad Nutr Diet.* 2017;117:1437-44.e2.

3. Horsley E. AAP Clinical Report on Lipid Screening in Children. *American Family Physician.* 2009;79(8):703–5.

4. National Health and Medical Research Council. Nutrient reference values for Australia and New Zealand. <https://www.eatforhealth.gov.au/nutrient-reference-values/nutrients>. Accessed 20 June 2024.
